# Analysis of Mitochondrial Function and Localisation during Human Embryonic Stem Cell Differentiation In Vitro

**DOI:** 10.1371/journal.pone.0052214

**Published:** 2012-12-19

**Authors:** Andrew B. J. Prowse, Fenny Chong, David A. Elliott, Andrew G. Elefanty, Edouard G. Stanley, Peter P. Gray, Trent P. Munro, Geoffrey W. Osborne

**Affiliations:** 1 The Australian Institute for Bioengineering and Nanotechnology, The University of Queensland, St. Lucia, Australia; 2 Monash Immunology and Stem Cell Laboratories, Monash University, Clayton, Australia; 3 Queensland Brain Institute, The University of Queensland, St. Lucia, Australia; 4 Murdoch Children’s Research Institute, The Royal Children’s Hospital, Parkville, Australia; University of Cincinnati, United States of America

## Abstract

Human embryonic stem cell (hESC) derivatives show promise as viable cell therapy options for multiple disorders in different tissues. Recent advances in stem cell biology have lead to the reliable production and detailed molecular characterisation of a range of cell-types. However, the role of mitochondria during differentiation has yet to be fully elucidated. Mitochondria mediate a cells response to altered energy requirements (e.g. cardiomyocyte contraction) and, as such, the mitochondrial phenotype is likely to change during the dynamic process of hESC differentiation. We demonstrate that manipulating mitochondrial biogenesis alters mesendoderm commitment. To investigate mitochondrial localisation during early lineage specification of hESCs we developed a mitochondrial reporter line, KMEL2, in which sequences encoding the green fluorescent protein (GFP) are targeted to the mitochondria. Differentiation of KMEL2 lines into the three germ layers showed that the mitochondria in these differentiated progeny are GFP positive. Therefore, KMEL2 hESCs facilitate the study of mitochondria in a range of cell types and, importantly, permit real-time analysis of mitochondria via the GFP tag.

## Introduction

Human embryonic stem cells (hESCs) are pluripotent cells that have the capacity to differentiate into multiple cell types of the adult body. These differentiating cell populations have a wide array of metabolic profiles and energy requirements. Mitochondria, as the energy powerhouses responsible for ATP production, play a pivotal role supplying the energy required during production and specification of all cell lineages. Characterisation of different cell types based on mitochondrial properties and localisation [1], [2] indicates the mitochondrial phenotype is an important consideration in the analysis of differentiated hESC progeny. However, recent studies suggest that IVF embryos used to derive hESCs frequently contain multiple mitochondrial DNA mutations [3], [4], [5]. In this context, it is noteworthy that mitochondrial disorders such as Friedreich’s Ataxia [6] or autosomal recessive spastic ataxia of Charlevoix-Saguenay [7] are often cell type specific. Given this association of mitochondrial dysfunction with human disease, cognisance of mitochondrial phenotype may be important for any future hESC based applications in regenerative medicine.

Mitochondria are maternally inherited intracellular organelles with a 16.6 kB genome [8]. The mitochondrial genome encodes for 13 of the 80 subunits of the electron transport chain (ETC) responsible for ATP production at the end point of oxidative phosphorylation. The mitochondrial genome also encodes 22 tRNAs and 2 rRNAs which, in a self-regulatory loop, are involved in the synthesis of the 13 mitochondrially derived subunits of the ETC (reviewed in [9]). Mitochondrial replication, inheritance, maintenance and function are controlled by an estimated 1500 nuclear encoded genes [10]. Two nuclear encoded proteins in particular, DNA polymerase gamma (*POLG*) and mitochondrial transcription factor A (*TFAM*) are involved in mitochondrial DNA replication and transcription [11]. Changes in expression levels of TFAM and POLG can be directly linked to variations in mitochondrial biogenesis and have been shown to be present at differing levels depending on the cell type, stage of differentiation and tissue of origin [12], [13].

HESCs have relatively few mitochondria and have poorly developed cristae [14], [15] with the cells predominantly relying on glycolysis for energy production [16], [17]. Mitochondria in hESCs appear punctate, are localised to the periphery of the nucleus (perinuclear) and have a restricted oxidative capacity [15], [18], [19]. Upon early differentiation, mitochondria undergo extensive distribution and branching throughout the cell [15], [18], [20] with a switch from glycolysis to oxidative phosphorylation [15], [18], [21]. This phenotype of mitochondrial localisation applies to multiple stem cell categories including adult, embryonic or induced pluripotent stem cells [5], [13], [15]. This redistribution of mitochondria in hESCs from a peri-nuclear localisation to a branched network precedes down regulation of typical hESC markers such as Oct-4 [20]. It has been suggested that the characteristics of hESC mitochondria and metabolism such as perinuclear localisation, low ATP content and a high metabolic rate could be used as a marker for “stemness” [3]. Indeed, there is increasing evidence that mitochondria and their associated patterns of metabolism and localisation are in fact inexorably linked to pluripotency maintenance [17] and that undifferentiated hESCs can suppress mitochondrial activity [13], [21]. Inhibition of mitochondrial function, or more specifically promoting glycolysis, enhances or maintains pluripotency with or without bFGF, respectively, and prevents early differentiation [20], [22]. In addition, recent reports on human induced pluripotent stem cells (hIPSC) show that during reprogramming, the properties of mitochondria and metabolism also revert to those of a more hESC-like phenotype. This included altered localisation of mitochondria, mitochondrially associated gene expression level, mitochondrial DNA content, ATP levels, lactate levels and oxidative damage [13], [16], [21].

While evidence of the important role mitochondria and glycolysis play in maintaining hESC pluripotency is emerging, there is currently little known about the role mitochondria play in hESC differentiation. It is known that mitochondria levels vary in different cell types [23], [24] and similarly their role in differentiation has been implicated in multiple human lineages including mesenchymal stem cells [25], [26], cardiac mesangioblasts [27] and embryonic stem cells [20]. Based on recent evidence, which indicates that hESC pluripotency status can be influenced by shifts in oxidative phosphorylation and glycolysis, we examined the molecular changes in mitochondrially associated genes in response to mitochondrial biogenesis agents. Furthermore, we show that actively promoting mitochondrial biogenesis and oxidative phosphorylation improves differentiation of hESC towards a primitive-streak like mesendoderm population. Finally, we developed a hESC line in which GFP fluorescently tags mitochondria from initial biogenesis to maturity, paving the way for future detailed study of mitochondrial changes as hESCs differentiate towards specific mature cell types. Collectively, our studies reaffirm the pivotal role played by mitochondria in early lineage commitment and provide new tools for investigation of this critical organelle during hESC differentiation.

## Materials and Methods

### Ethics Statement

HESC line MEL2 was previously derived on mouse embryonic fibroblast (MEF) feeder layers under approval from the Australian National Health and Medical Research Council (Licence No. 309709).

### Tissue Culture

All mammalian tissue culture reagents described here were from Life Technologies (Carlsbad, CA, USA) unless otherwise stated. The *MIXL1* reporter line has been described [28]. All lines were provided by Stem Core Queensland (Australian Stem Cell Centre) and routinely maintained as manually passaged cultures on MEF feeder layers as previously described [29]. Prior to experiments, cells were either grown in bulk culture or adapted to single cell passage as previously described [30], [31].

### Immunofluorescence

Cells were fixed in ethanol and stained overnight at 4°C for markers of differentiation and pluripotency according to [32]. Primary antibodies used were mouse IgG_1_ anti-mitochondria (clone 113-1, 2 µg/mL), mouse IgG_1κ_ anti- Oct-4 (2 µg/mL), mouse IgG_3_ anti-SSEA-4 (2 µg/mL), mouse IgG_1_ anti-Tra-2-49 (2 µg/mL), mouse IgG_2a_ anti-TG30 (1 µg/mL), mouse IgG_2a_ anti-α-fetoprotein (AFP, 2 µg/mL), rabbit IgG anti-nestin (5 µg/mL) and mouse IgG_1_ anti-MAP-2 (5 µg/mL), mouse IgG_1_ anti-β3-tubulin, (all from Merck Millipore). Isotype specific secondary antibodies were used conjugated to Alexa fluor 488, 568, 633 or 647. Secondary antibodies were used at 1 µg/mL. Nuclei were counter stained with DAPI at 1 µg/mL. Fluorescence was visualised using an EVOS_fl_ inverted microscope (Advanced Microscopy Group) or an Inverted LSM 510 Meta (Zeiss Microscopy, Germany). Images and fluorescence profile data were generated using Image J (v1.41). For live cell imaging, nuclei were stained with Hoechst 33342 (1 µg/mL) and mitochondria with LDS-751 (0.2 µg/mL), Mitotracker deep-red (Life Technologies, according to manufacturer instructions) for 15 minutes at 37°C. Mitosox red was used at 5 µM for 30 mins at 37°C.

### Flow cytometry

Expression of TG30 was determined by flow cytometry using a BD LSRII flow cytometer, as previously described [32]. Dead cells were discriminated using 10 µg/mL propidium iodide and cell doublets and clumps using forward and side scatter characteristics [33]. Flow data were analysed on Eclectic and Lucid (Version 2.0, Walter and Eliza Hall Institute for Medical Research) or CFlow Sampler (v1.0.264.15, Accuri Cytometers). Live cell images of LDS-751 stained hESC were taken using an Amnis Image Stream Cytometer.

### Mesendoderm Specific Differentiation

Mesendoderm lineage detection was conducted using the *MIXL1* reporter line [28] with protocols previously shown to promote cardiac mesoderm formation [34]. Briefly, the day before differentiation, cells were harvested with TrypLE SELECT and seeded at 60–80% confluency on a flask coated with 1×10^4^/cm^2^ irradiated MEFs. The next day, cells were harvested and seeded at 3000 cells/well of a 96 well, non-treated U-bottom plate (Nalge Nunc International) in APEL media with growth factors, BMP4 (20 ng/ml, R&D Systems), Activin A (20 ng/ml), VEGF (40 ng/ml), SCF (30 ng/ml) and Wnt3a (100 ng/ml, all from PeproTech) and set up as spin embryoid bodies [34]. Relative MIXL1 expression was measured on day 3 based on GFP fluorescence using flow cytometry on an Accuri C6 cytometer.

### Mitochondrial Biogenesis

To test the effect of mitochondria biogenesis agents, SNAP (*S*-nitroso-*N*-acetylpenicillamine), AICAR (5-Aminoimidazole-4-carboxamide 1-β-D-ribofuranoside) and Metformin were added to MIXL1 embryoid bodies or 2D feeder free cultures (Geltrex™ surface coating and StemPro® media) at 0–500 µM and cultured for 3 days. Prior to RNA extraction, hESC were harvested with TrypLE SELECT and seeded at 100,000 cells per well of a 24 well plate coated with Geltrex™ in StemPro® media. The cells were grown for 2 days in the presence of SNAP, AICAR and Metformin from 0–500 µM before harvesting for RNA as below.

### Quantitative PCR (qPCR)

The full protocol used closely adheres to recent guidelines on conducting and reporting on qPCR results [35]. Briefly, RNA was extracted from hESC as single cell cultures using the Qiagen RNeasy RNA extraction kit (Qiagen). Genomic DNA was removed using Turbo DNA-free kit according to the manufacturer’s instructions (Life Technologies). One microgram of DNA free RNA was converted to cDNA using Life Technologies’s Superscript III cDNA synthesis kit and oligo (dT)_20_ primers. CDNA was diluted 1∶10 before qPCR. Primer sequences used for qPCR can be found in Table 1. QPCR was performed using an Applied Biosystems 7500 Fast ThermoCycler and SYBR® Green Master Mix with 1 step of 95°C for 20 seconds followed by 40 cycles of 95°C for 3 seconds/60°C for 30 seconds. Primer-product specificity was confirmed by the presence of one peak in a step wise melt curve analysis and visualisation of bands on 1.5% agarose gels. Standard StemPro® cultures were used as the control sample and all genes referenced to human β-actin mRNA using the Pfaffl method [36] for POLG and TFAM. β-actin was used as the reference gene [32]. All experiments and qPCR runs were conducted in triplicate.

**Table 1:** qPCR primer sequences.

Primer	Sequence	Product size (base pairs)
TFAM Fwd-115	CCG AGG TGG TTT TCA TCT GT	203
TFAM Rev-317	TCC GCC CTA TAA GCA TCT TG	
POLG Fwd-1490	CCC ATG AGG TTT TCC AGC	127
POLG Rev-1616	AGG TAA CGC TCC CAG TTC	

### Transfection

The full transfection protocol can be found in Methods S1. Briefly, MEL2 cells, p32 (manual dissection) +3 (bulk culture) +11 (single cells) were treated with Rock inhibitor (Y27632, 10 µM final concentration, Sigma Aldrich, St Louis, MO, USA) for 1 hour prior to transfection. Detached cells were resuspended at 1×10^6^ cells/100 µL in Human Stem Cell Nucleofector® Solution 2 (Lonza) containing 2ug/100 µL of the commercially available DNA plasmid pEF/*myc*/mito/GFP (Life Technologies). Aside from a neomycin selection cassette, the plasmid contained a GFP sequence tagged to a mitochondrial import sequence under the control of the EF1α promoter. Cells were transfected using program B-016 on a Nucleofector® II cuvette device (Lonza). Transfection efficiency using this program set was measured to be approximately 50% with 78% viable cells post transfection (not shown). Transfected cells were transferred to one well of a 12 well plate pre-coated with Geltrex™ and grown in StemPro®. Transfected cells were allowed to recover for 24hrs before selection in G418. The mitochondrial reporter line was designated KMEL2.

### HESC *In Vitro* Differentiation

To assess the ability of KMEL2 to differentiate, KSR media was exchanged for DMEM without bFGF and supplemented with 10% foetal bovine serum (FBS). Cells were also treated with Retinoic acid (10 µg/mL, Sigma Aldrich), BMP4 (40 ng/mL, R & D Systems, Minneapolis, MN, USA) or Activin A (40 ng/mL, PeproTech) for up to 10 days to promote germ layer specific differentiation. For neural specific differentiation, KMEL2 cells were grown feeder free on Geltrex™ to 60% confluence. Media was changed to KSR supplemented with 100 ng/mL bFGF, 5 µM dorsomorphin, 10uM SB431542 and grown for 6 days with media changed every other day. Cell clumps were treated with collagenase IV to form embryoid bodies and transferred to suspension culture in KSR with bFGF, SB431542 and dorsomorphin for a further 3 days. Embryoid bodies where then grown for up to 30 days prior to plating on mouse laminin (10 µg/cm^2^, Sigma-Aldrich) coated dishes to allow for neural outgrowth.

### Karyotype Analysis

Karyotyping analysis was conducted on KMEL2 at passage 7 post transfection as previously described [37]. 15 metaphases per sample were analysed and images taken at a resolution of 400bphs. Karyotype analysis was conducted by Sullivan Nicolaides Pathology, Taringa, Australia.

### Statistical Analysis

Statistical analysis was conducted using two-tailed paired student’s t-tests or two-way ANOVA with replication. P values <0.05 were considered significant. All experiments were performed with a minimum of 3 biological replicates and a minimum of 3 inter-experiment replicates.

## Results

### Mitochondrial Biogenesis Agents Impact on hESC Differentiation

**Figure 1:**
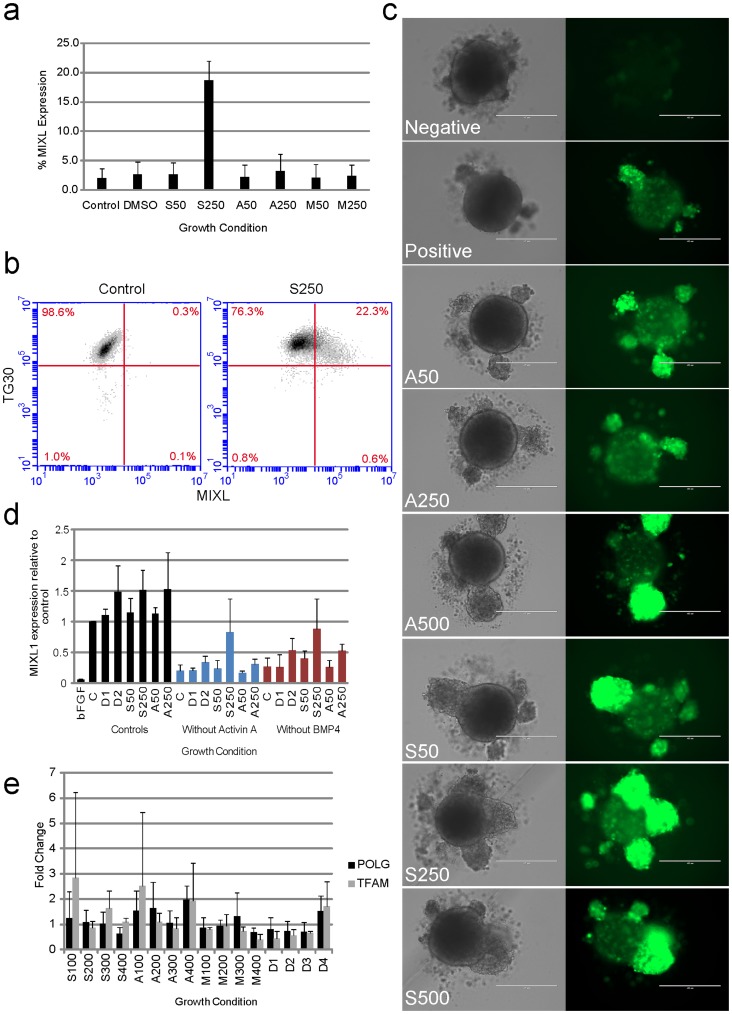
Mitochondrial biogenesis agents enhance MIXL1 expression in differentiating hESC. (a) SNAP can induce MIXL1 expression in StemPro® 2D cultures independent of BMP4 addition (p<0.05, n = 4). (b)The pluripotency marker TG30 is down regulated in cells positive for mesendoderm marker MIXL1 at day3 post 250 µM SNAP treatment. (c) Differentiation to early mesoderm (day 3) is enhanced in 3D cultures by addition of mitochondrial biogenesis agents. Scale bars are 200 µM. (d) 250 µM SNAP can partially rescue MIXL1 expression on removal of Activin A or BMP4. Control samples were treated according to ([44], black bars) or cultured without BMP 4 (red bars) or without Activin A (blue bars). (e) Mitochondrially associated gene expression is highly variable after SNAP and AICAR treatment. S, SNAP; A, AICAR; M, metformin; POLG, polymerase gamma; TFAM, mitochondrial transcription factor A; numbers represent µM concentrations of reagents used; D, DMSO without biogenesis agents was added as controls in equal volumes to treated samples.

Attenuation of mitochondrial function and promotion of glycolysis has been used to promote increased expression of pluripotency markers and inhibit differentiation [20]. Conversely, we sought to investigate whether promotion of mitochondrial biogenesis (and subsequently an increase in oxidative phosphorylation) would influence differentiation of hESC towards early mesoderm. We investigated three chemical agents, SNAP, AICAR and metformin with known effects on mitochondrial biogenesis and cell differentiation in human and other mammalian species [25], [38], [39], [40]. To determine if increasing mitochondrial biogenesis had any impact on differentiation, independent of factors to promote differentiation, MIXL1 cells were grown for 3–4 days on Geltrex coated plates in hESC maintenance media StemPro® with or without biogenesis agents. At day 4, 18.7±3.2% of cells treated with 250 µM SNAP were positive for MIXL1 expression (Figure 1a, p<0.05, n = 3, compared to untreated controls) and demonstrated down regulation of the pluripotency marker TG30 (Figure 1b) and SSEA-4 (not shown). Concentrations of SNAP at 250 µM or above had detrimental effects on cell number and mitochondrial membrane potential as assessed by JC-1 staining (Figure S1). Neither AICAR nor metformin increased the percentage of MIXL1 positive cells above untreated controls (Figure 1a). To determine if any biogenesis agents could increase MIXL1 positive cells during cardiogenic mesoderm induction, spin embryoid bodies were differentiated using APEL medium [34] and growth factors BMP4, Activin A, VEGF and SCF. Increasing concentrations of both SNAP and AICAR increased the percentage of MIXL1 positive cells 17.33±11.72 (p<0.05) and 13.41±13.4% (p>0.05) respectively above controls (Figure S2) as well as the relative level of MIXL1 expression within the cells (Figure 1c). In order to determine a positive impact of biogenesis agents on MIXL1 expression, embryoid bodies were formed in the presence of biogenesis agents diluted in DMSO with and without the key growth factors for differentiation, BMP4 and Activin A. As expected removal of either BMP4 or Activin A significantly impacted on MIXL1 expression (Figure 1d). However, MIXL1 expression was partially restored in cultures lacking BMP4 or Activin A by 250 µM SNAP, but not the DMSO control or AICAR treated samples (Figure 1d and S2 and 3). Mitochondrial biogenesis in hESC was measured by the expression of *POLG* and *TFAM*, nuclear encoded genes required for mitochondrial DNA replication and transcription respectively (for review see [11]). No treatment yielded a significant change in expression of *POLG* or *TFAM* (p>0.05). However both Metformin and the DMSO controls exhibited a trend in down regulation of each gene (Figure 1e). In contrast, SNAP and AICAR had a highly variable effect on gene expression and trended towards increasing expression of *TFAM* and *POLG*.

### Generating a Human Embryonic Stem Cell Mitochondrial Reporter Line: KMEL2

**Figure 2:**
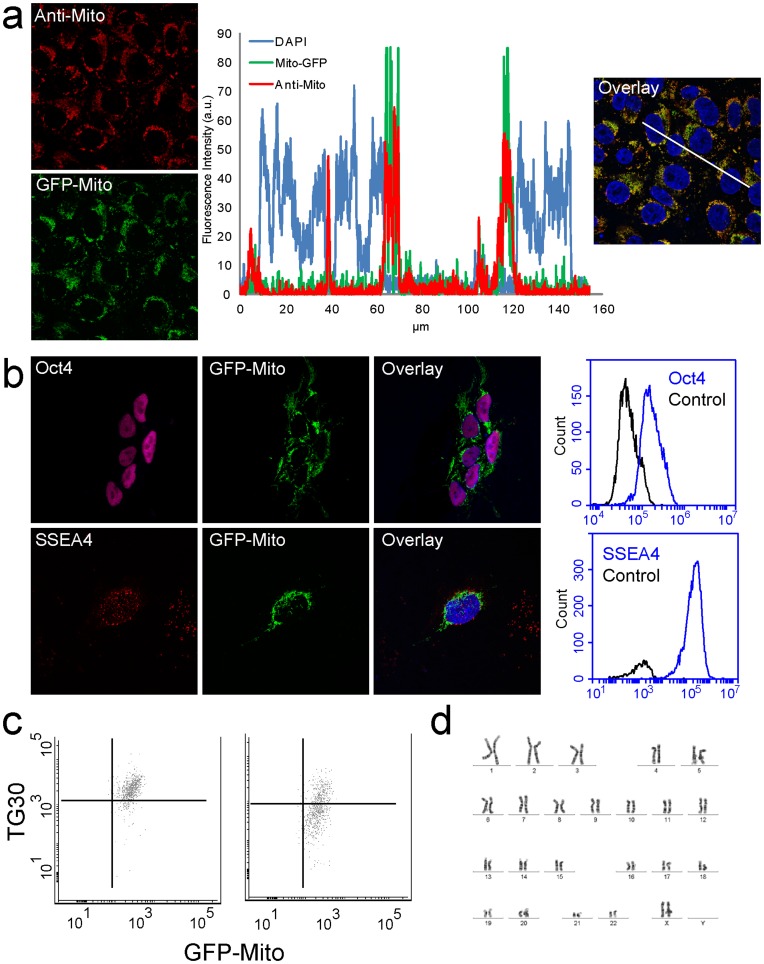
Generation of a mitochondrial reporter line, KMEL2. Cells were transfected with a plasmid encoding mitochondrially targeted GFP expressed under the control of an EF1α promoter. (a) Subcellular localisation of mitochondrially targeted GFP overlaps with structures detected by an anti-mitochondrial antibody. Fluorescence intensities for each fluorophore were measured along the 160 µm line shown in the overlay image. (b) Pluripotency marker expression is not effected by mitochondrially targeted GFP. GFP localised to the mitochondria is co-expressed with pluripotency markers Oct-4 and SSEA4. Images are 150 µm wide. Co-expression of GFP and pluripotency markers was confirmed by flow cytometry. Histograms show GFP positive cells also express Oct-4 and SSEA-4. (c) GFP intensity is not lost during down regulation of cell surface pluripotency marker TG30. (d) KMEL2 cells have a normal karyotype.

MEL2 hESCs transfected with pEF/*myc*/mito/GFP were selected using G418 over a three week period. The resulting GFP positive hESC line was designated KMEL2. The mitochondrial localization of GFP in KMEL2 cells was confirmed with an anti-mitochondrial antibody (Figure 2a) and staining with Mitosox red (Figure S5). Measuring fluorescence intensity along a line profile shows a precise overlap of the GFP and mitochondrial antibody signals indicating co-localisation (Figure 2a). The transgenic cell line retained expression of the pluripotency markers, Oct-4, SSEA-4 (Figure 2b), TG30 and Tra-2-49 (Figure S4) between 5 and 10 passages post-transfection. In addition, KMEL2 cells maintained a normal karyotype (Figure 2d). Flow cytometric analysis showed that GFP expression remained robust at day (d) 4 of differentiation while expression of the pluripotency markers TG30 (Figure 1c) and SSEA-4 (not shown) were down regulated. Thus, GFP expression is maintained during early hESC differentiation.

### The Fluorochrome LDS-751 Localises to Mitochondria in hESC

**Figure 3:**
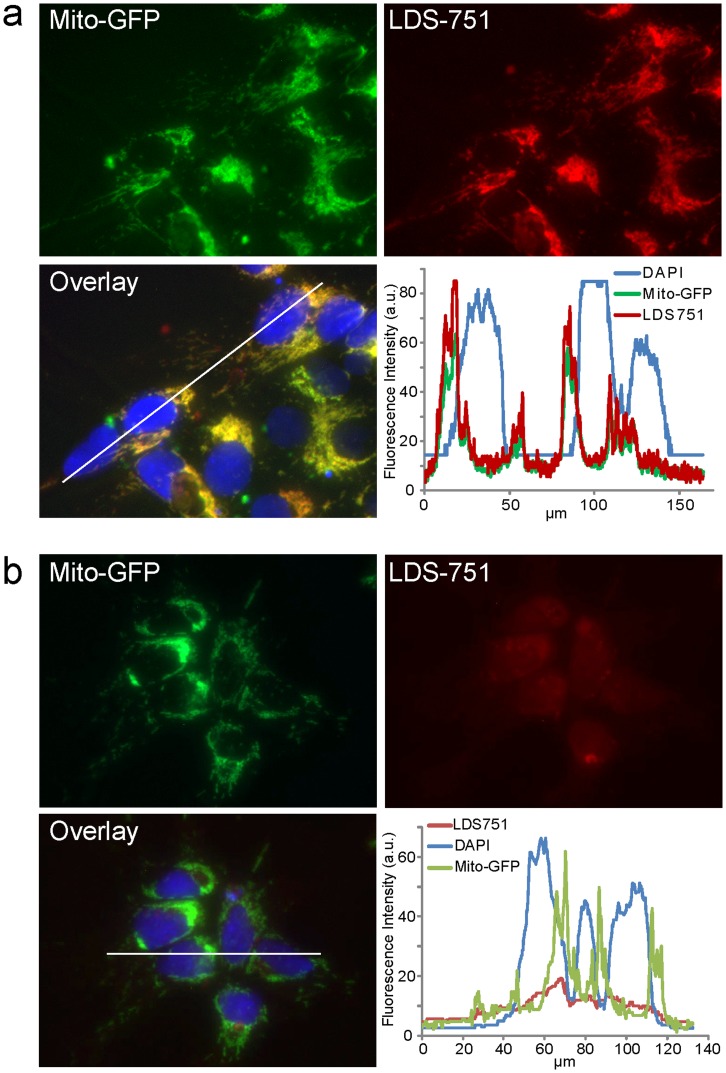
LDS-751 stains human embryonic stem cell mitochondria based on membrane potential. (a) LDS-751 is co-localised with GFP in the KMEL2 mitochondria reporter line and does not overlap with the nucleus. Fluorescence intensities for each fluorophore were measured along the 160 µm line shown in the overlay image and plotted as distance vs intensity. (b) Mitochondria specific staining is lost when treated with a mitochondrial membrane depolarising agent valinomycin. Line profile analysis demonstrates LDS-751 no longer localised to the mitochondria after blocking mitochondrial membrane potential. The line profile in the overlay image represents 140 µm.

To further validate the use of KMEL2 in live tracking of hESC mitochondria, we used flow based image analysis to confirm mitochondrial GFP localisation. We initially used LDS-751 as a nuclear counter stain, because it has no significant spectral overlap with GFP. However, in LDS-751 stained KMEL2 cells, significant co-localisation of LDS-751 with GFP was observed (Figure S5). This suggests LDS-751 does not stain the nucleus in hESC. This was confirmed in a 2-dimensional format using DAPI as a nuclear stain. LDS-751 did not co-localise with the nuclear stain DAPI and, instead, LDS-751 overlapped exclusively with GFP imported to the mitochondria (Figure 3a). In addition, LDS-751 co-localised exclusively with mitosox red (Figure S5). Depolarisation of mitochondrial membranes with valinomyocin inhibited the localisation of LDS-751 to mitochondria (Figure 3b). Mitochondrial localisation of LDS-751 has previously been reported in mouse fibroblasts and monocytes and, as for hESC, was dependent on polarised mitochondrial membranes [41]. Thus, LDS-751 can be used as a tool for tracking mitochondria in cultured cells.

### Mitochondrial Localisation During Differentiation of All Three Germ Layers

**Figure 4:**
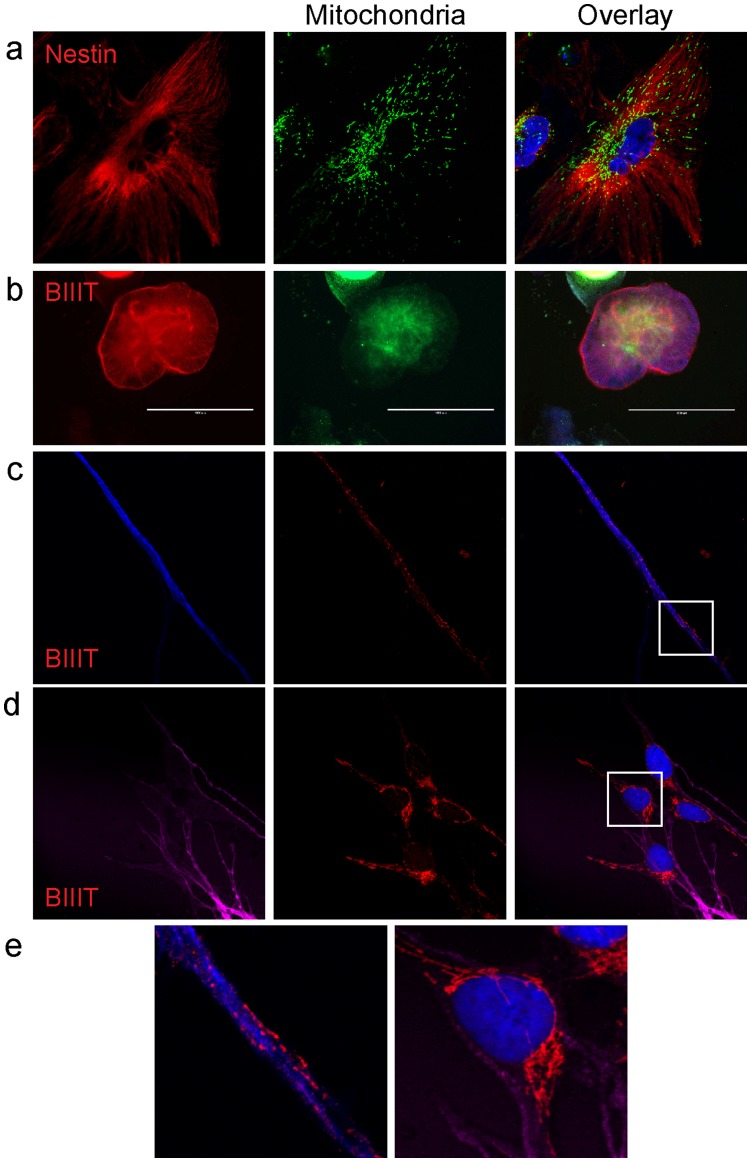
Mitochondrial localisation during neural lineage differentiation. Neural lineage specific differentiation showing KMEL2 positive for (a) Nestin and (c-e) β-III-tubulin. β-III-tubulin positive cells show expanded localisation of mitochondria through dendritic outgrowths (c and e). βIIIT, β-III-tubulin. Scale bars in (b) are 1000 µm. All other images are 150 µm wide. Enlarged images in (e) are shown in the boxed regions of (c) and (d).

**Figure 5:**
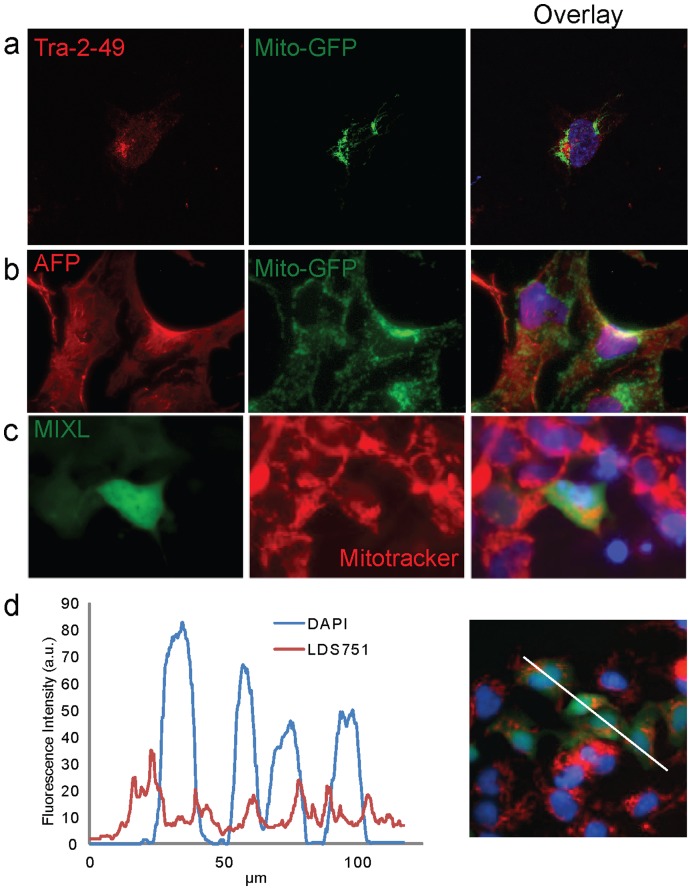
Variable mitochondrial localisation during lineage specific differentiation. (a) Mitochondria in hESC are localised near the nucleus. (b) Mitochondria in AFP positive endoderm lineage cells. Mitochondria in AFP positive cells display a granular, dispersed localisation through the whole cell. (c and d) Mitochondria in MIXL1 positive cells (Mesendoderm) display a densely packed, perinuclear localisation based on MitoTracker far red (c) and LDS-751 (d) staining. AFP, alpha fetoprotein. Images (a-c) are 150 µm wide. Line profile in (d) represents 120 µm.

During hESC differentiation significant changes occur in mitochondrial metabolism, morphology and energy output (oxidative phosphorylation vs. glycolysis) [15], [18], [20]. However, little information is available on localisation and morphology of mitochondria during lineage specific differentiation. We used the KMEL2 reporter line and LDS-751 to track mitochondria during retinoic acid driven neuroectoderm differentiation. Consistent with previous data [2], [15], mitochondria in hESC prior to differentiation were closely localised to the periphery of the nucleus in dense clusters shown with both KMEL2 and LDS-751 (Figure 2b, 3b and 5a). In contrast, KMEL2 derived Nestin and MAP2C positive cells had mitochondria dispersed throughout the cell in granular and thread-like patterns (Figure 4a and Figure S4), as previously reported in adult cells from the neural lineage [42], [43]. Embryoid bodies plated on laminin after 30 days of neural specific differentiation show GFP (through anti-GFP antibody binding) localisation to mitochondria in β-III-tubulin positive cells (Figure 4b-e) confirmed by co-staining with an anti-mitochondrial antibody (not shown). Further, mitochondrial clusters could be identified in dendritic outgrowths positive for β-III-tubulin (Figure 4c and e).

Differentiation to the endoderm lineage was identified with AFP and FOXA2 staining (Figure 5b and S4). Similar to mitochondrial localisation in Nestin positive cells, AFP positive cells contained mitochondria dispersed throughout the cell in a granular formation with a limited amount of perinuclear mitochondrial clustering.

In order to observe mitochondria during the formation of cardiac competent mesoderm a reporter line for the mesendoderm marker MIXL1 [28] was used in conjunction with published protocols to drive the induction of cardiogenic mesoderm [44]. Cells positive for MIXL1 on d3-d4 of differentiation were stained for mitochondria using either LDS-751 or Mito-tracker Deep Red. The mitochondrial localisation in MIXL1 positive cells is similar to undifferentiated hESC with mitochondria densely localised to the nuclear periphery (Figure 5c).Line profile analysis of fluorescence intensities for LDS-751 and DAPI confirmed a tight clustering of mitochondria around the nucleus (Figure 5d).

## Discussion

In order to investigate the role mitochondria play in regulating the balance between pluripotency and lineage commitment we developed a mitochondrial reporter hESC cell line that expresses a mitochondrially localized GFP, KMEL2. Importantly, we demonstrate that GFP expression is maintained in derivatives from all germ layers when KMEL2 hESC differentiate. The KMEL2 hESC line also facilitated the identification of mitochondrial biogenic reagents that promote differentiation of primitive mesendoderm.

### Tools for *in vivo* Mt Analysis

In this study we developed two approaches to identifying and tracking mitochondrial localisation in hESC and their differentiated progeny. Firstly, we developed a mitochondrial reporter hESC cell line that produced a GFP construct tagged to a mitochondrial import sequence as has been shown for multiple cell types [45], [46]. The reporter line, dubbed KMEL2, showed co-localisation of GFP with specific antibodies to mitochondria (Figure 2a), expressed pluripotency markers Oct-4 and SSEA-4 (Figure 2b) and retained a normal karyotype post transfection (Figure 2d). KMEL2 is particularly useful for tracking mitochondrial localisation and structural alterations during differentiation. Mitochondrial tracking may be important in therapeutic applications, for example the clumping of mitochondria in cellular prolongations during hESC neural differentiation is a characteristic phenotype of mitochondrial disorders such as ARSACS [7]. Secondly, we show that in hESC, LDS-751 co-localised specifically with GFP in the KMEL2 line and showed no significant overlap with the nuclear stain DAPI (Figure 3a). Whilst LDS-751 has been previously used as a nuclear marker [47] we show that mitochondrial localisation in hESCs is dependent on mitochondrial membrane polarisation as treatment with the depolarising agent valinomycin blocked mitochondrial specific staining (Figure 3b).

### Promotion of Oxidative Phosphorylation Enhances Differentiation

Mitochondrial biogenesis is controlled by peroxisome proliferator-activated receptor-γ coactivator-1α (*PGC-1α*), *NRF-1* and *TFAM* [11]. Metformin and AICAR are known activators of AMP-activated protein kinase (AMPK) [39] which in turn increases the production of PGC-1α. PGC-1α co-activates the transcription of *TFAM* [48], a direct regulator of mitochondrial DNA transcription and replication. SNAP is a nitric oxide (NO) donor, also known to increase expression of mitochondrial biogenesis genes such as *TFAM* and *POLG* however its mode of action is to directly activate *PGC-1α* [49] thus indirectly increasing mitochondrial biogenesis. The fold changes (1.5 to 3) we observed in the mitochondrial biogenesis regulators *TFAM* and *POLG*, although variable, concurred with published results [15], [21], [39], [50]. In addition, SNAP and AICAR displayed a trend of increasing levels of TFAM and POLG suggesting increased mitochondrial biogenesis.

We observed that SNAP induced mitochondrial biogenesis in cytokine free StemPro media lead to an increased production of MIXL1^+^ cells. In contrast, neither Metformin nor AICAR induced expression in these conditions. Conversely, in differentiating embryoid bodies both SNAP and AICAR increased the number of MIXL1 positive cells by approximately 15% compared to untreated controls (Figure S2). Furthermore, in the absence of the key differentiation factors BMP4 or ACTIVIN A, SNAP was able to partially restore MIXL1 expression in embryoid bodies. However, AICAR could not substitute for these cytokines in the embryoid body assay. This suggests that SNAP and AICAR may have different modes of action in promoting differentiation. For example, SNAP may induce differentiation [38] through either mitochondrial biogenesis or an as yet unknown pathway, while AICAR may not induce differentiation but may inhibit pluripotency thereby improving the general differentiation of the cells regardless of lineage. A possible confounding factor is that embryoid bodies without BMP4 and ACTIVIN A were smaller compared to controls (Figure S3). Nevertheless, further testing of differentiation efficiency in combinatorial titrations of AICAR or SNAP in lineage specific differentiation protocols is needed to precisely define the role of mitochondria in differentiation.

### Conclusion

Normal cell function requires coordinated communication between the nucleus and mitochondria for efficient transcription of ETC components. An essential part of this communication is the localisation of intracellular “messengers” to particular areas of the cell, as is evident with peri-nuclear localisation of mitochondria in hESC prior to differentiation. We have generated novel methods for the visualization of mitochondria in hESC during differentiation and investigated the role of mitochondria in lineage specific differentiation to mesoderm. These traceable mitochondria provide a powerful means of investigating the changes in mitochondria during differentiation of varying cell lineages and facilitate the analysis of the impact of biogenesis on differentiation trajectories. Finally, mitochondrial characteristics may provide a means of further classifying differentiated stem cell progeny for use in therapeutic applications.

## Supporting Information

Figure S1 **Treatment with SNAP lowers hESCs numbers and mitochondrial membrane potential.** a) MIXL1 cells were seeded into 24 well plates and treated for 24hrs with biogenesis agents indicated or DMSO as control. Cells were grown feeder free on Geltrex coated plates. On day 3 cells were harvested and counted using a standard haemocytometer. Error bars are +/−SD of n = 3 biological replicates. b) MIXL1 and Nkx2.5 cells were seeded into 24 well plates and treated for 24hrs with biogenesis (50 or 250uM) agents indicated or DMSO as control. Cells were grown feeder free on Geltrex coated plates. On day 3 cells were harvested and treated with 5uM JC-1 for 15mins at RT. Bars represent relative cell numbers with low membrane potential. Error bars are +/−SD of n = 3 biological replicates. S = SNAP, A = AICAR, M = Metformin.
(PDF)

Figure S2 **MIXL1 expression post treatment with biogenesis agents.** a) AICAR and SNAP at 500 µM in the presence of BMP4 and Activin A increase MIXL1 expression relative to controls. b) Individual replicate data represented in part “a” expressed as MIXL expression relative to control. c) Raw data of MIXL expression expressed as percentage positive for MIXL expression. n/a = test not performed, Dead = cell death prohibited analysis, A = AICAR, S = SNAP, concentrations listed as A50, A250 etc represent µM, S = SNAP, A = AICAR, M = Metformin.
(PDF)

Figure S3 **MIXL expression in hESCs treated with biogenesis agents in the absence of Activin A or BMP4.** C = control (all growth factors VEGF, SCF, BMP4 and Activin A), A- = Differentiation without Activin A, B- = Differentiation without BMP4, A50 and A250 = AICAR concentrations of 50 and 250 µM, S50 and S250 = SNAP concentrations of 50 and 250 µM.
(PDF)

Figure S4 **Lineage specific marker expression in KMEL2.** a) KMEL2 cells express embryonic stem cell marker Tra-2-49. b) KMEL2 cells express embryonic stem cell marker TG30. Histograms represent flow cytometry data demonstrating GFP positive cells express pluripotency markers (blue line) above negative controls (black line). c) Mitochondria show a dispersed localisation in MAP2C positive cells. d) KMEL2 cells differentiated towards the endoderm lineage express FOXA2.
(PDF)

Figure S5 **Mitochondria visualisation in KMEL2.** a) LDS-751 (pink) co-localises with GFP in KMEL2 cells (green). Images taken on an Amnis image stream. b) GFP, LDS-751 and Mitosox red co-localise in KMEL2 cells. c) Profile analysis of fluorescence intensity for each mitochondrial marker demonstrates overlapping of peak signals. Line of profile is shown in overlay image from “b”.
(PDF)

Methods S1 **Early images of KMEL2 selection post transfection.** MEL2 hESCs were transfected to label mitochondria as described in Supplementary Method S1. Scale bars are 200 µm.
(PDF)
