# Supplementary material for: Analysis of Mitochondrial Function and Localisation during Human Embryonic Stem Cell Differentiation In Vitro
Source: PLoS One. 2012 Dec 19;7(12):e52214. doi: 10.1371/journal.pone.0052214 (PMC3526579; doi:10.1371/journal.pone.0052214)
Supplement: Figure S1 — Treatment with SNAP lowers hESCs numbers and mitochondrial membrane potential. a) MIXL1 cells were seeded into 24 well plates and treated for 24hrs with biogenesis agents indicated or DMSO as control. Cells were grown feeder free on Geltrex coated plates. On day 3 cells were harvested and counted using a standard haemocytometer. Error bars are +/−SD of n = 3 biological replicates. b) MIXL1 and Nkx2.5 cells were seeded into 24 well plates and treated for 24hrs with biogenesis (50 or 250uM) agents indicated or DMSO as control. Cells were grown feeder free on Geltrex coated plates. On day 3 cells were harvested and treated with 5uM JC-1 for 15mins at RT. Bars represent relative cell numbers with low membrane potential. Error bars are +/−SD of n = 3 biological replicates. S = SNAP, A = AICAR, M = Metformin. (PDF) [file pone.0052214.s001.pdf]

# Supplementary Figure S1

a

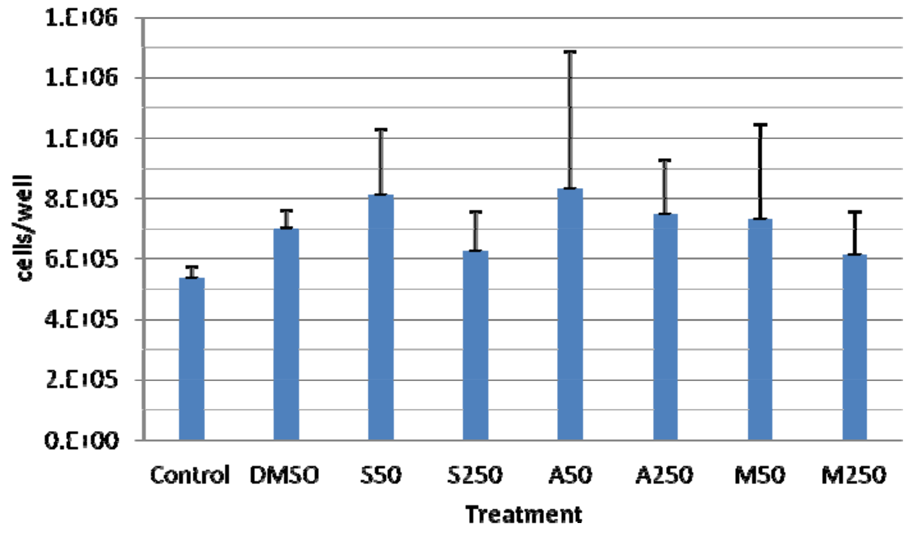


b

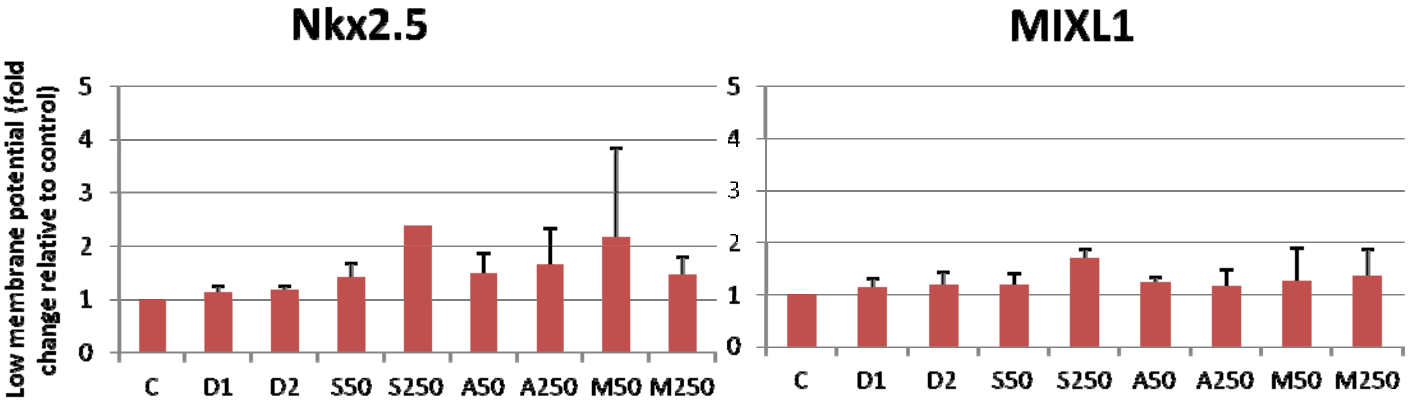


**Supplementary Figure S1**. Treatment with SNAP lowers hESCs numbers and mitochondrial membrane potential. a) MIXL1 cells were seeded into 24 well plates and treated for 24hrs with biogenesis agents indicated or DMSO as control. Cells were grown feeder free on Geltrex coated plates. On day 3 cells were harvested and counted using a standard haemocytometer. Error bars are +/-SD of n=3 biological replicates. b) MIXL1 and Nkx2.5 cells were seeded into 24 well plates and treated for 24hrs with biogenesis (50 or 250uM) agents indicated or DMSO as control. Cells were grown feeder free on Geltrex coated plates. On day 3 cells were harvested and treated with 5uM JC-1 for 15mins at RT. Bars represent relative cell numbers with low membrane potential. Error bars are +/-SD of n=3 biological replicates. S = SNAP, A = AICAR, M = Metformin.
