# Supplementary material for: Analysis of Mitochondrial Function and Localisation during Human Embryonic Stem Cell Differentiation In Vitro
Source: PLoS One. 2012 Dec 19;7(12):e52214. doi: 10.1371/journal.pone.0052214 (PMC3526579; doi:10.1371/journal.pone.0052214)
Supplement: Figure S2 — MIXL1 expression post treatment with biogenesis agents. a) AICAR and SNAP at 500 µM in the presence of BMP4 and Activin A increase MIXL1 expression relative to controls. b) Individual replicate data represented in part “a” expressed as MIXL expression relative to control. c) Raw data of MIXL expression expressed as percentage positive for MIXL expression. n/a = test not performed, Dead = cell death prohibited analysis, A = AICAR, S = SNAP, concentrations listed as A50, A250 etc represent µM, S = SNAP, A = AICAR, M = Metformin. (PDF) [file pone.0052214.s002.pdf]

Supplementary Figure S2

a

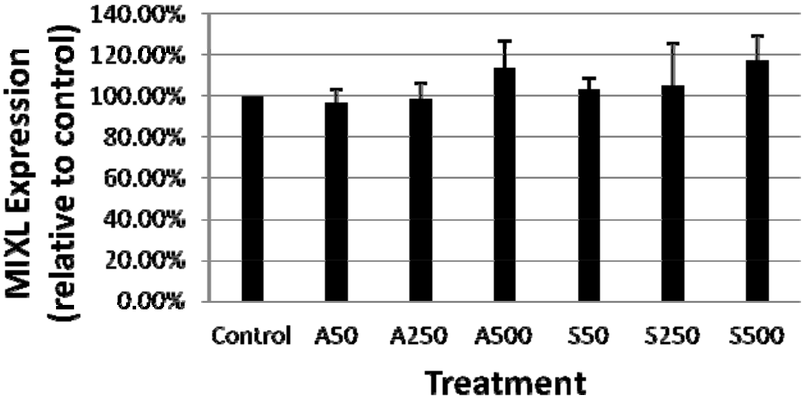

b

Relative to positive control									
**Replicate**	**1**	**2**	**3**	**4**	**5**	**6**	**7**	**Average**	**SD**
Control	100.00%	100.00%	100.00%	100.00%	100.00%	100.00%	100.00%	100.00%	0.00%
A50	n/a	85.82%	95.70%	102.99%	101.68%	98.77%	94.95%	96.65%	6.18%
A250	n/a	86.83%	90.72%	102.40%	104.70%	102.19%	103.03%	98.31%	7.54%
A500	116.02%	100.19%	133.38%	101.87%	dead	115.57%	dead	113.41%	13.40%
S50	n/a	101.29%	113.72%	101.53%	100.22%	101.78%	99.21%	102.96%	5.36%
S250	n/a	109.75%	120.58%	102.39%	104.25%	125.68%	67.79%	105.07%	20.42%
S500	110.60%	128.02%	126.43%	104.29%	dead	dead	dead	117.33%	11.72%

c

	PBS as diluent				With DMSO as diluent				
	19/11/2010	8/11/2010	26/09/2010	29/10/2010	2/03/2010	27/04/2010	16/03/2010	**Average**	**SD**
bFGF Control	1.71%	0.79%	1.35%	3.41%	3.20%	4.80%	3.20%	3%	0.011287
Pos Control	78.61%	56.50%	43.74%	87.36%	89.40%	73.20%	89.10%	74%	0.176427
A50		48.49%	41.86%	89.97%	90.90%	72.30%	84.60%	71%	0.046881
A250		49.06%	39.68%	89.46%	93.60%	74.80%	91.80%	73%	0.066327
A500	91.20%	56.61%	58.34%	88.99%		84.60%		76%	0.194903
S50		57.23%	49.74%	88.70%	89.60%	74.50%	88.40%	75%	0.052962
S250		62.01%	52.74%	89.45%	93.20%	92%	60.40%	75%	0.065549
S500	86.94%	72.33%	55.30%	91.11%				76%	0.158354

Supplementary Figure S2. **MIXL1 expression post treatment with biogenesis agents .**

a) AICAR and SNAP at 500µM in the presence of BMP4 and Activin A increase MIXL1 expression relative to controls. b) Individual replicate data represented in part "a" expressed as MIXL expression relative to control. c) Raw data of MIXL expression expressed as percentage positive for MIXL expression. n/a = test not performed, Dead = cell death prohibited analysis, A = AICAR, S = SNAP, concentrations listed as A50, A250 etc represent µM, S = SNAP, A = AICAR, M = Metformin.
