# Supplementary material for: Analysis of Mitochondrial Function and Localisation during Human Embryonic Stem Cell Differentiation In Vitro
Source: PLoS One. 2012 Dec 19;7(12):e52214. doi: 10.1371/journal.pone.0052214 (PMC3526579; doi:10.1371/journal.pone.0052214)
Supplement: Figure S3 — MIXL expression in hESCs treated with biogenesis agents in the absence of Activin A or BMP4. C = control (all growth factors VEGF, SCF, BMP4 and Activin A), A- = Differentiation without Activin A, B- = Differentiation without BMP4, A50 and A250 = AICAR concentrations of 50 and 250 µM, S50 and S250 = SNAP concentrations of 50 and 250 µM. (PDF) [file pone.0052214.s003.pdf]

Supplementary Figure S3

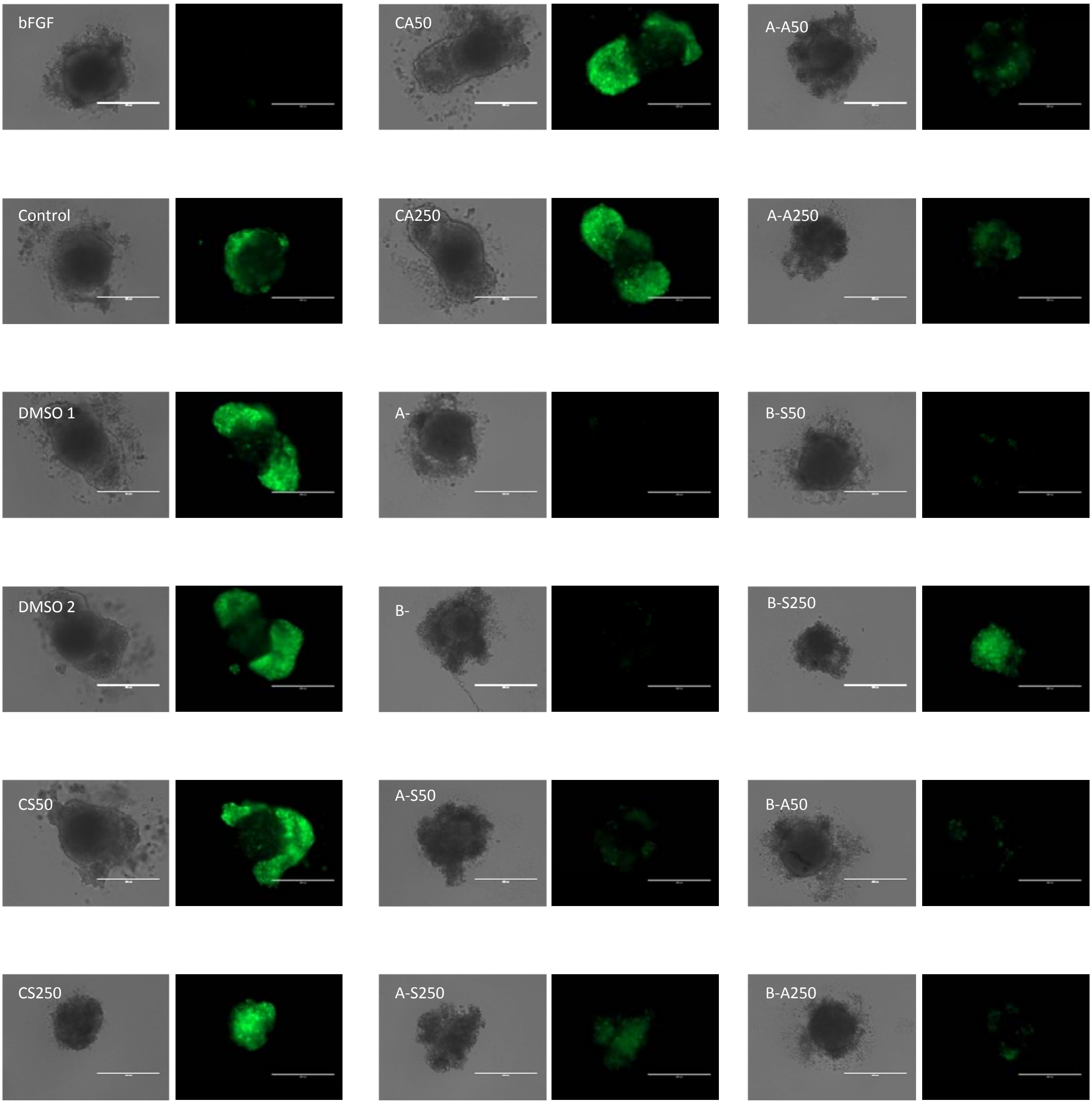

Supplementary Figure S3. **MIXL expression in hESCs treated with biogenesis agents in the absence of Activin A or BMP4**. C = control (all growth factors VEGF, SCF, BMP4 and Activin A), A- = Differentiation without Activin A, B- = Differentiation without BMP4, A50 and A250 = AICAR concentrations of 50 and 250µM, S50 and S250 = SNAP concentrations of 50 and 250µM.
