# Supplementary material for: Analysis of Mitochondrial Function and Localisation during Human Embryonic Stem Cell Differentiation In Vitro
Source: PLoS One. 2012 Dec 19;7(12):e52214. doi: 10.1371/journal.pone.0052214 (PMC3526579; doi:10.1371/journal.pone.0052214)
Supplement: Figure S4 — Lineage specific marker expression in KMEL2. a) KMEL2 cells express embryonic stem cell marker Tra-2-49. b) KMEL2 cells express embryonic stem cell marker TG30. Histograms represent flow cytometry data demonstrating GFP positive cells express pluripotency markers (blue line) above negative controls (black line). c) Mitochondria show a dispersed localisation in MAP2C positive cells. d) KMEL2 cells differentiated towards the endoderm lineage express FOXA2. (PDF) [file pone.0052214.s004.pdf]

Supplementary Figure S4

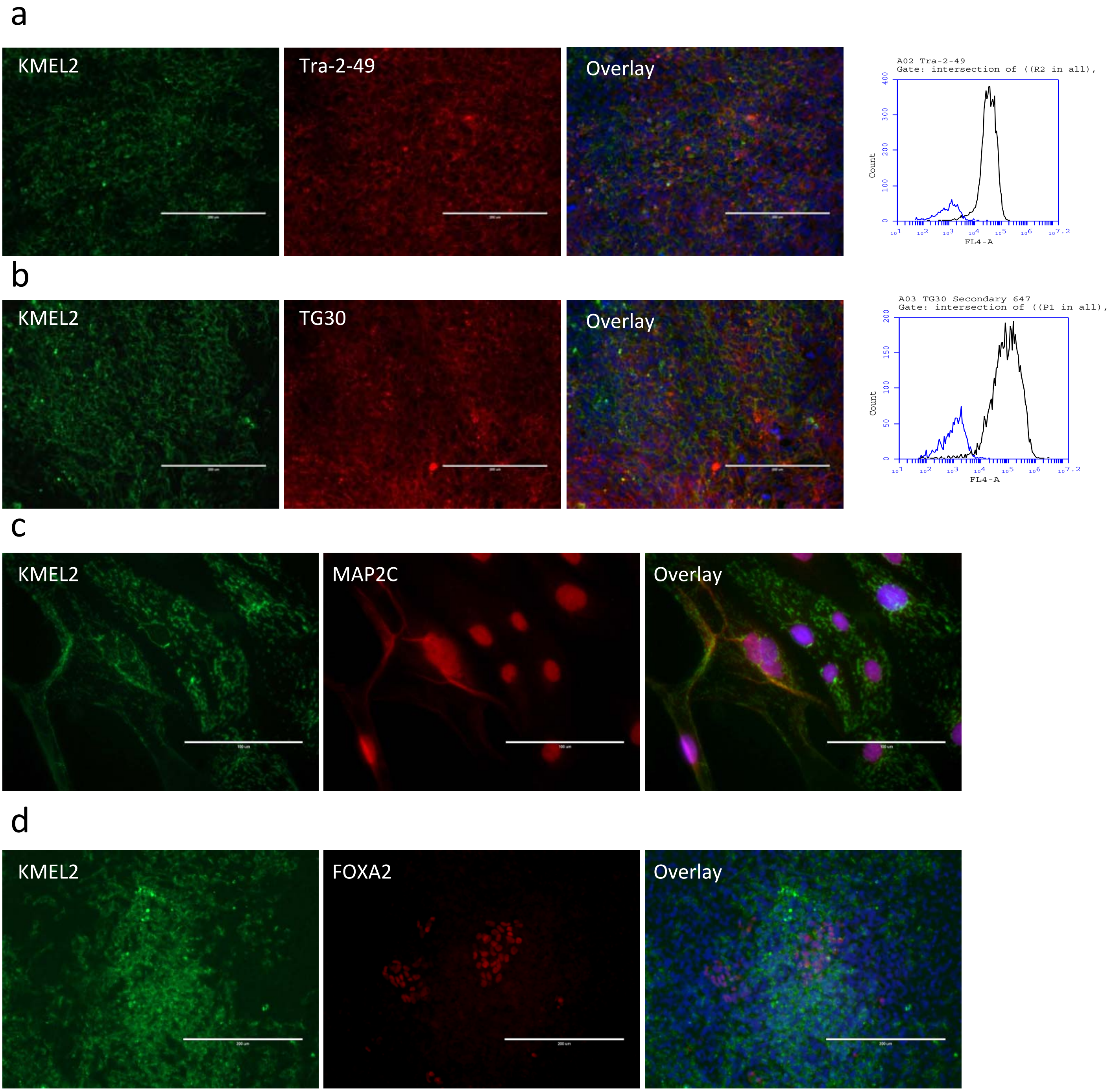

Supplementary Figure S4. Lineage specific marker expression in KMEL2. a) KMEL2 cells express embryonic stem cell marker Tra-2-49. b) KMEL2 cells express embryonic stem cell marker TG30. Histograms represent flow cytometry data demonstrating GFP positive cells express pluripotency markers (blue line) above negative controls (black line). c) Mitochondria show a dispersed localisation in MAP2C positive cells. d) KMEL2 cells differentiated towards the endoderm lineage express FOXA2.
