# Supplementary material for: Analysis of Mitochondrial Function and Localisation during Human Embryonic Stem Cell Differentiation In Vitro
Source: PLoS One. 2012 Dec 19;7(12):e52214. doi: 10.1371/journal.pone.0052214 (PMC3526579; doi:10.1371/journal.pone.0052214)
Supplement: Figure S5 — Mitochondria visualisation in KMEL2. a) LDS-751 (pink) co-localises with GFP in KMEL2 cells (green). Images taken on an Amnis image stream. b) GFP, LDS-751 and Mitosox red co-localise in KMEL2 cells. c) Profile analysis of fluorescence intensity for each mitochondrial marker demonstrates overlapping of peak signals. Line of profile is shown in overlay image from “b”. (PDF) [file pone.0052214.s005.pdf]

Supplementary Figure S5

a

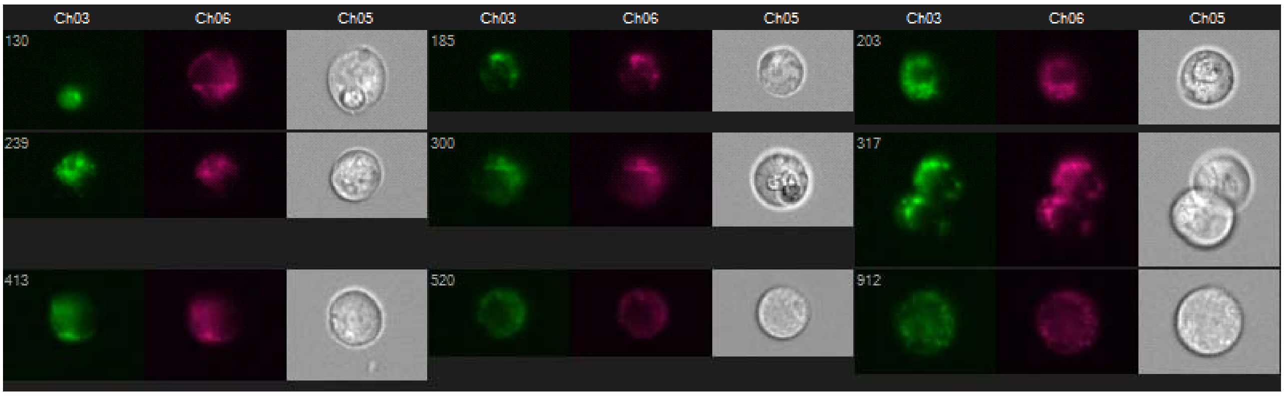

b

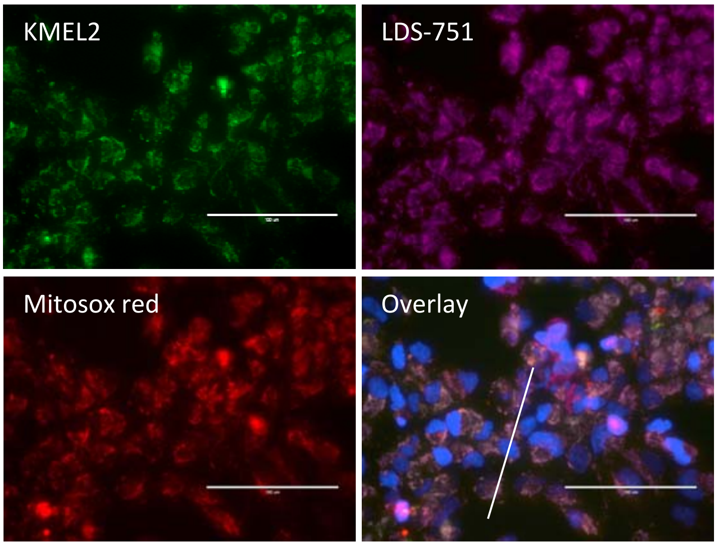

c

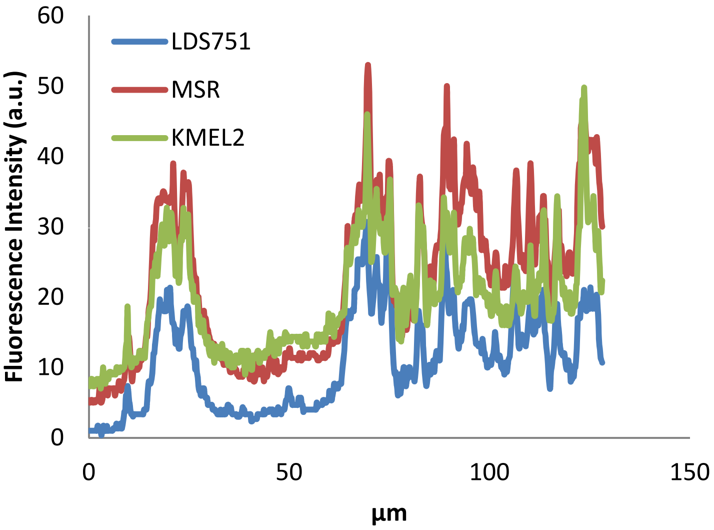

Supplementary Figure S5. Mitochondria visualisation in KMEL2. a) LDS-751 (pink) co-localises with GFP in KMEL2 cells (green). Images taken on an Amnis image stream. b) GFP, LDS-751 and Mitosox red co-localise in KMEL2 cells. c) Profile analysis of fluorescence intensity for each mitochondrial marker demonstrates overlapping of peak signals. Line of profile is shown in overlay image from “b”.
