# Supplementary material for: Analysis of Mitochondrial Function and Localisation during Human Embryonic Stem Cell Differentiation In Vitro
Source: PLoS One. 2012 Dec 19;7(12):e52214. doi: 10.1371/journal.pone.0052214 (PMC3526579; doi:10.1371/journal.pone.0052214)
Supplement: Methods S1 — Early images of KMEL2 selection post transfection. MEL2 hESCs were transfected to label mitochondria as described in Supplementary Method S1. Scale bars are 200 µm. (PDF) [file pone.0052214.s006.pdf]

Supplementary Method S1: Transfection

Twelve well plates were coated with Geltrex (1:200, 700µL/well) for 1 hour prior to transfection. MEL2 cells, p32 (manual dissection) +3 (bulk culture) +11 (single cells) were treated with Rock inhibitor (Y27632, 10µM final concentration,. Sigma Aldrich, St Louis, MO, USA) for 1 hour prior to transfection. One millilitre of StemPro® media was added to each well and incubated at 37°C, 5% CO_2 immediately prior to transfection. Single cells were detached using cell dissociation buffer. Cells were resuspended at $1x10^{6}$ cells/100µL in Human Stem Cell Nucleofector® Solution 2 (Lonza, Waverley, Australia) containing 2ug/100uL of the DNA plasmid pEF/*myc*/mito/GFP (see supplementary material for plasmid structure). Plasmids were linearised using EcoRI prior to transfection. Cells were transfected using program B-016 on a Nucleofector® II cuvette device (Lonza, see Supplementary Material for transfection optimisation). Five hundred microlitres of StemPro® media was added to the cells and the entire solution transferred to one well of the pre-coated 12 well plate. Transfected cells were allowed to recover for 24hrs before selection in G418. Selection in G418 was conducted in gradual increments from 25µg/mL-300µg/mL over a three week period.

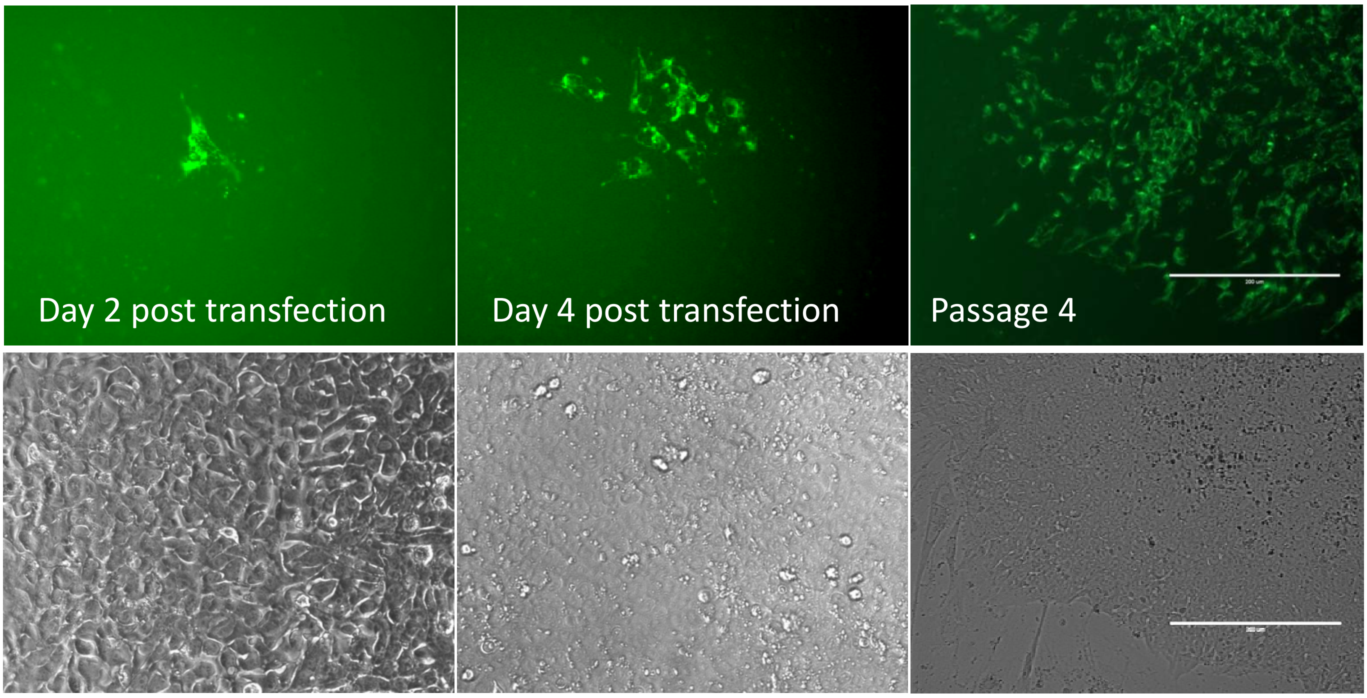

Supplementary Method S1. Early images of KMEL2 selection post transfection. MEL2 hESCs were transfected to label mitochondria as described in Supplementary Method S1. Scale bars are 200µm.
